# Public versus private trauma intensive care unit outcomes: A retrospective cohort study

**DOI:** 10.4102/jcmsa.v4i1.411

**Published:** 2026-09-18

**Authors:** Mariska Els, Naadiyah Laher, Megan Lubout, Maeyane S. Moeng

**Affiliations:** 1 Department of Surgery, School of Clinical Medicine, Faculty of Health Sciences, University of the Witwatersrand, Johannesburg, South Africa

**Keywords:** outcome assessment, hospital mortality, severity of illness index, healthcare sector, intensive care unit, trauma

## Abstract

**Background:**

Trauma remains a leading cause of morbidity and mortality in low- and middle-income countries (LMICs), with South Africa experiencing a high burden of intentional and high-energy injuries. Disparities in critical care resources between the public and private healthcare sectors have been reported, but comparative trauma intensive care unit (ICU) outcomes remain incompletely described.

**Methods:**

This retrospective cohort study compared adult trauma patients admitted to public and private healthcare sector ICUs in Johannesburg between July 2022 and June 2023. The primary outcome was 30-day in-hospital mortality. Secondary outcomes included injury characteristics, severity of illness and factors associated with mortality. Descriptive statistics and multivariate logistic regression were used for data analysis.

**Results:**

A total of 453 trauma patients were included (private ICU *n* = 209; public ICU *n* = 244), with male predominance in both cohorts. Private ICU patients were older, more frequently sustained blunt trauma, and had longer ICU stays. Public ICU patients were younger and more likely to sustain penetrating trauma (68.4% vs 14.4%; *p* < 0.001). Thirty-day mortality did not differ significantly (private 8.6% vs public 11.9%; *p* = 0.255), despite greater illness and injury severity among private ICU patients. Greater severity of illness and injury independently predicted mortality, while public ICU admission was associated with increased adjusted odds of mortality.

**Conclusion:**

Despite differences in injury profiles, patient mortality was similar in both healthcare sectors. Illness severity remains the prevailing determinant of outcome, underscoring the importance of severity scoring systems and potential system-level factors in LMICs’ trauma care. Level of evidence: Level III; prognostic and epidemiological study.

**Contribution:**

This study provides comparative trauma ICU outcomes data from the public and private healthcare sectors in Johannesburg, highlighting the role of illness severity and healthcare sector infrastructure on patient mortality and informing trauma critical care in LMICs.

## Introduction

Trauma is a major global cause of mortality and disability, disproportionately affecting low- and middle-income countries (LMICs) where injury-related deaths surpass those from non-communicable diseases.^1^ Young people, particularly economically active populations are affected, amplifying the socio-economic consequences of injury.^2,3,4,5^ South Africa experiences exceptionally high rates of trauma related to interpersonal violence, firearm-related injury and road traffic collisions, with a substantial proportion of these patients requiring critical care admission.^2,3,4,5,6,7^ Recent data from major trauma centres in Johannesburg confirm that penetrating trauma, particularly interpersonal violence, continues to account for the majority of trauma presentations in South African urban hospitals.^8^ Despite this burden, detailed data describing trauma-specific intensive care unit (ICU) outcomes in the South African context remain limited.

Access to timely surgical and critical care services is a key determinant of survival in severely injured patients, making intensive care an essential component of current trauma systems. A 30-day mortality is widely used as a benchmark outcome in trauma research; however, comparative analyses examining outcomes across differing healthcare system structures are scarce, particularly within LMIC settings. In resource-constrained environments, ICU admission decisions often require careful triage, reinforcing the importance of objective prognostic tools to support equitable and effective allocation of critical care resources.^9^

South Africa’s dual healthcare system provides a unique context in which to evaluate trauma ICU outcomes. The public sector serves the majority of the population within a resource-limited environment, while the private sector operates under a distinct funding and infrastructure model with relatively greater access to advanced critical care resources.^10^ Understanding whether these structural differences translate into measurable differences in trauma ICU outcomes may provide valuable insight for trauma system development and resource allocation, locally and in comparable LMICs.^4,11^

The primary objective of this study was to compare 30-day in-hospital mortality between public and private trauma ICUs. These two ICUs were chosen by the authors because the same trauma surgeons involved in patient care work at both facilities. Secondary objectives were to compare injury and physiological severity profiles and to identify factors independently associated with mortality. By characterising the patient profiles and outcomes across these two healthcare sectors, this study may inform critical care resource planning and contribute to the growing body of trauma system research in middle-income countries.

## Research methods and design

### Study design and setting

This retrospective cohort study was conducted at two dedicated trauma ICUs in Johannesburg, South Africa: Charlotte Maxeke Johannesburg Academic Hospital (public sector), a tertiary-level academic referral centre, and a Level 1 private trauma hospital. Both institutions are accredited Level I trauma centres, providing consultant-led trauma services with 24-h access to definitive operative care, subspecialty surgical services, advanced imaging and critical care. These centres were intentionally selected as both ICUs are surgeon-led, with the same group of trauma surgeons providing both operative and critical care management. In the absence of dedicated intensivists, the trauma surgeons function as the primary intensivists throughout the patient’s ICU admission.

### Study population

Adult trauma patients (≥18 years) admitted to either ICU between 01 July 2022 and 30 June 2023 were eligible for inclusion. The study period was selected to represent a contemporary post-coronavirus disease 2019 (COVID-19) period, after trauma volumes and patterns of injury had largely returned to routine practice. A consecutive 12-month period was chosen to minimise seasonal variation in trauma presentations while providing an adequate sample of eligible patients for analysis. Both direct admissions and inter-facility transfers were included. Exclusion criteria comprised patients with burn injuries and patients with incomplete or missing data. Admission to the public trauma ICU was reserved for patients requiring organ support because of limited critical care capacity. Admission criteria for the private ICU were determined according to institutional practice.

### Data collection

Data were retrospectively extracted from ICU records and trauma databases. Variables included demographics, healthcare sector (public or private), mechanism of injury, Injury Severity Score (ISS), Revised Trauma Score (RTS), Shock Index (SI), Acute Physiological Chronic Health Evaluation (APACHE II) score, operative interventions, organ support requirement, development of nosocomial sepsis and 30-day in-hospital mortality. For the purpose of this study, nosocomial sepsis was defined as a positive microbiological culture (blood, urine, sputum or intraoperative fluid specimen) obtained during the hospital admission that resulted in the initiation of antimicrobial therapy. Pre-hospital data and referral pathways were not captured in this study.

### Outcomes

The primary outcome was 30-day in-hospital mortality. Secondary outcomes included comparison of severity scores and identification of mortality predictors.

### Statistical analysis

Continuous variables are reported as medians with interquartile ranges and compared using the Wilcoxon rank-sum test. Categorical variables are reported as frequencies and percentages and compared using chi-square or Fisher’s exact tests. Logistic regression analysis was used for univariable and multivariable analyses. Stata version 17 (StataCorp LLC, College Station, TX, United States) was used, with a *p*-value < 0.05 considered statistically significant.

### Ethical considerations

Ethical clearance to conduct this study was obtained from the University of the Witwatersrand Human Research Ethics Committee (Medical) (No: M240143 M240628-A-0001). Permission was also obtained from each hospital’s ethics committee, database gatekeepers. Patient consent was waived because of the retrospective nature of this study.

## Results

### Patient demographics and injury characteristics

A total of 453 patients were analysed (Figure 1, Table 1). There was a male predominance in both cohorts, with a higher proportion of female patients in the private ICU (18.2% vs 5.7%; *p* < 0.001). Private ICU patients were significantly older and had longer ICU stays. Penetrating trauma (stab wounds and gunshot wounds) predominated in the public ICU, whereas blunt trauma was more common in the private ICU (Table 1, Figure 2).

**FIGURE 1:**
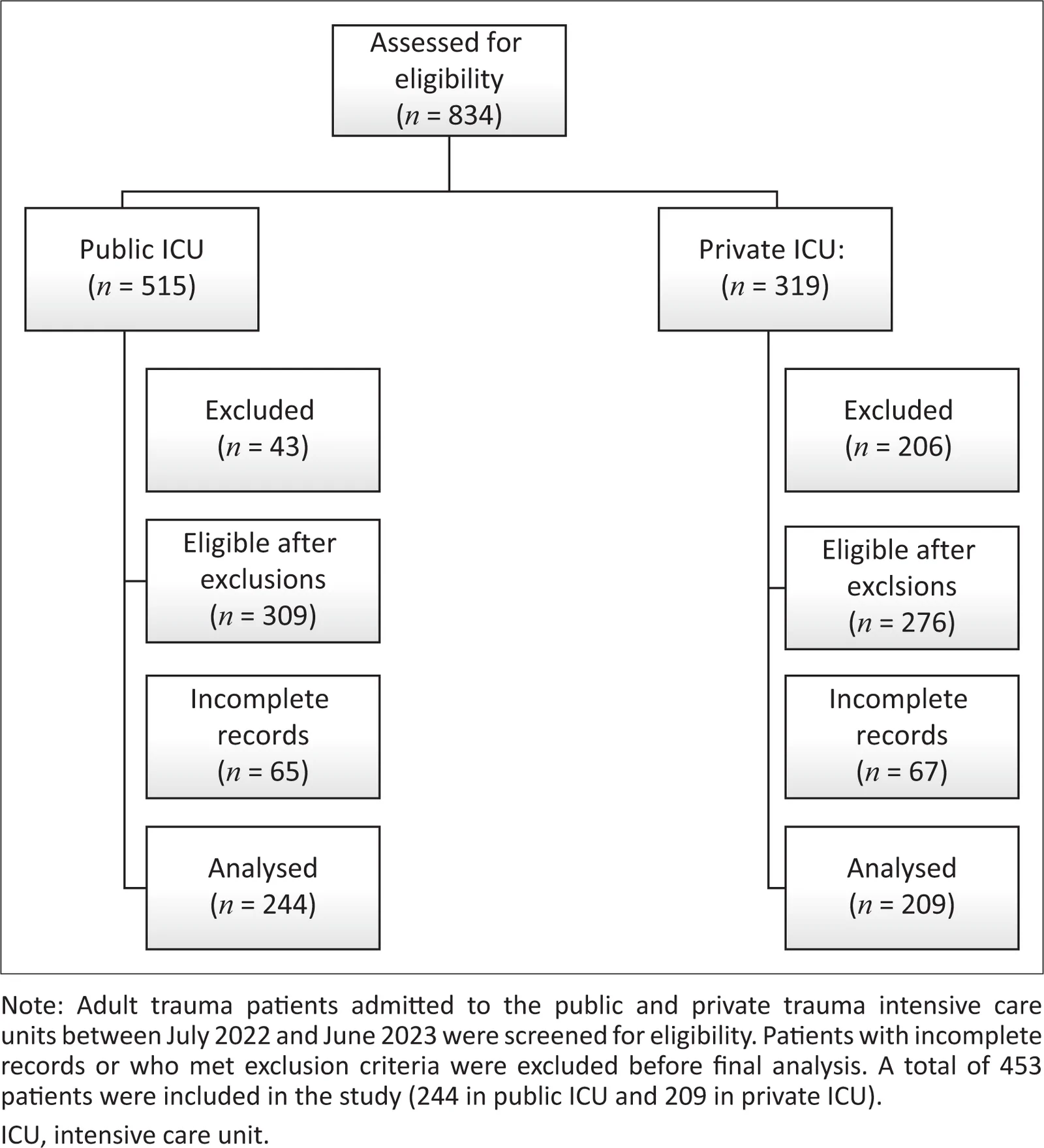
Flow diagram of patient selection.

**FIGURE 2:**
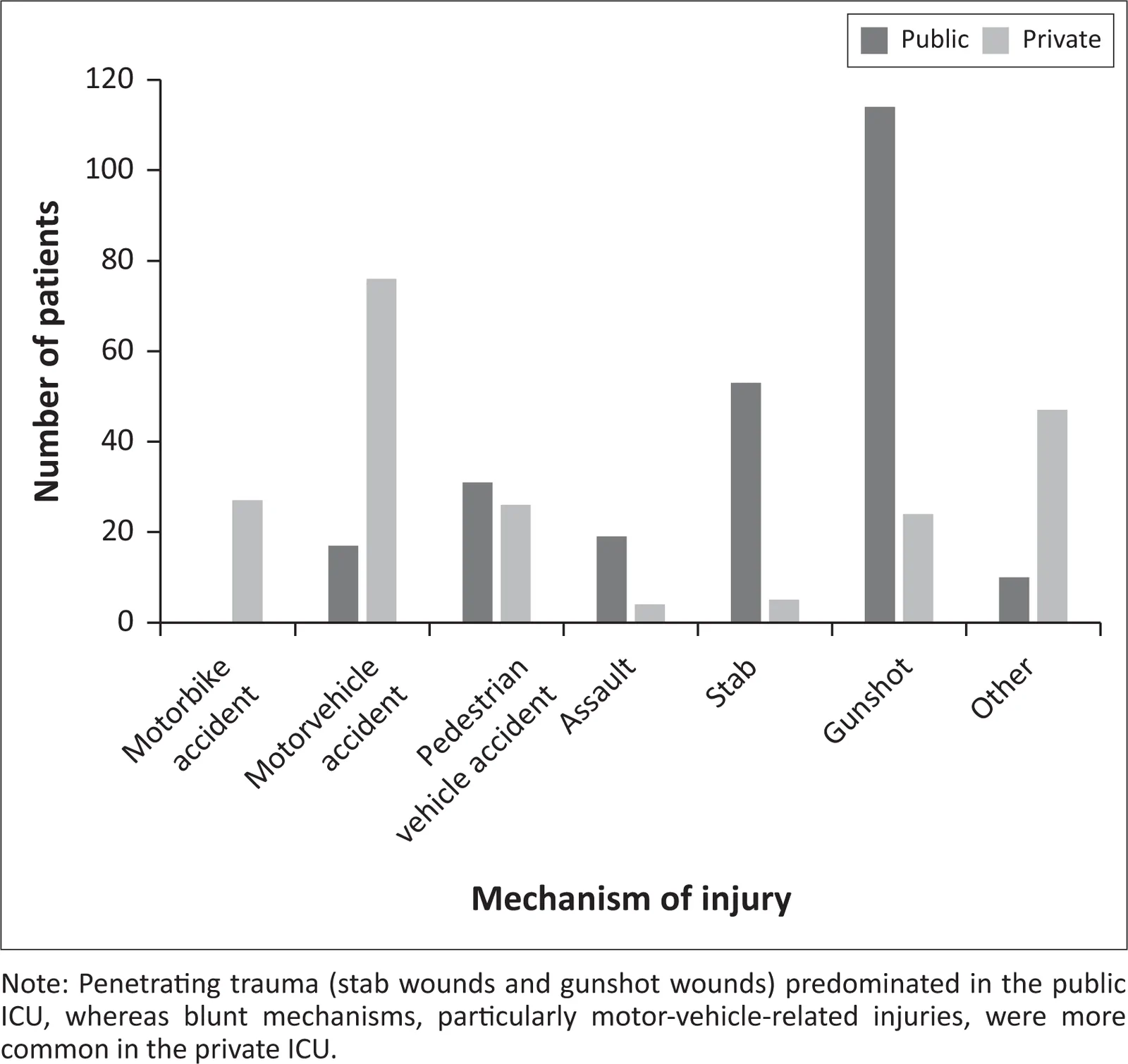
Distribution of mechanisms of injury among patients admitted to the public and private trauma intensive care units.

**TABLE 1:** Patient demographic characteristics.

Variable	Total (*n*)	Private TICU				Public TICU				*p*-value
		*n*	%	Median	IQR	*n*	%	Median	IQR	
**Gender**										
Male	401	171	81.8	-	-	230	94.3	-	-	-
Female	52	38	18.2	-	-	14	5.7	-	-	< 0.0010
**Age**	-	-	-	39.00	31–53	-	34.0	27–40.5	-	< 0.0001
**Mechanism of injury**										
Blunt	210	143	68.4	-	-	67	27.5	-	-	-
Penetrating (stab and gunshot wounds)	197	30	14.4	-	-	167	68.4	-	-	< 0.0010
Other	46	36	17.2	-	-	10	4.1	-	-	-
**Length of stay [days]**	-	-	-	10.00	5–26	-	-	5	3–11	< 0.0001
30-day mortality	47	18	8.6	-	-	29	11.9	-	-	0.2550
**ICU and trauma scores**										
APACHE II	-	-	-	18.00	12–23	-	-	16	11.5–21	0.0090
ISS	-	-	-	24.00	16–34	16	-	16	12–22	< 0.0001
RTS	-	-	-	4.10	4.1–7.6	-	-	8	5–8	< 0.0001
SI	-	-	-	0.77	0.62–1.02	-	-	0.85	0.69–1.05	0.0280
**Organ support**										
Overall	-	172	82.0	-	-	202	83.0	-	-	-
Ventilation	-	160	76.0	-	-	199	82.0	-	-	0.1910
Inotropes	-	123	58.9	-	-	95	38.9	-	-	< 0.0010
Dialysis	-	26	12.0	-	-	18	7.0	-	-	0.7000

APACHE II, acute, physiological chronic health evaluation II; ICU, intensive care unit; ISS, injury severity score; SI, shock index; TICU, trauma intensive care unit; IQR, interquartile range.

### Severity scores

Patients in the private ICU had significantly higher APACHE II and ISS values and lower RTS values, indicating greater physiological and anatomical injury severity (Table 1, Figure 3). Shock Index values differed modestly between sectors.

**FIGURE 3:**
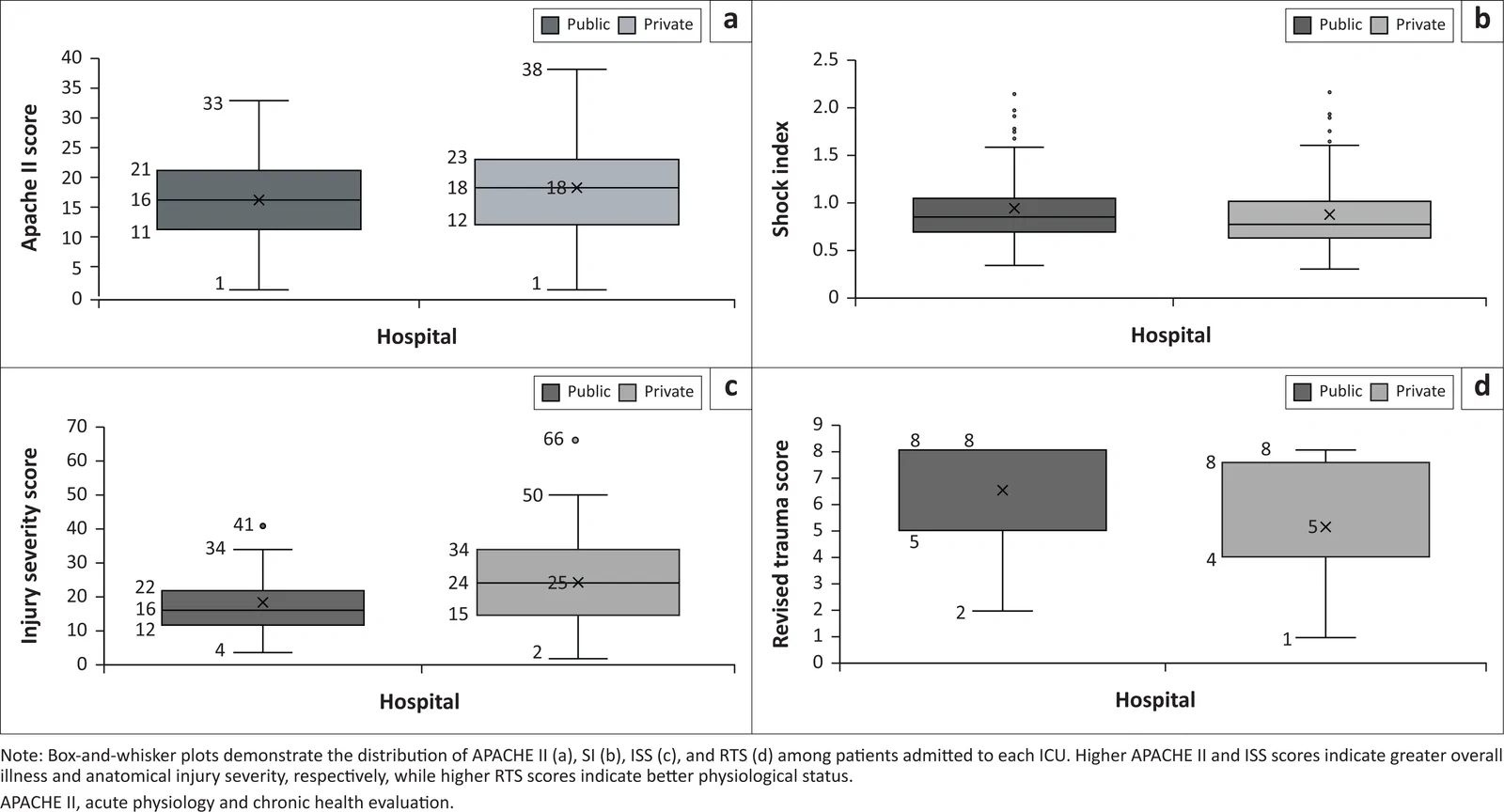
Comparison of (a) APACHE II, (b), Shock index (SI) (c), Injury Severity Score (ISS), and (d) Revised Trauma Score (RTS), between public and private trauma intensive care units.

### Organ support and interventions

Organ support requirements are summarised in Table 1. Rates of mechanical ventilation were similar between sectors, while inotropic support and renal replacement therapy were more frequently utilised in the private ICU. Nosocomial sepsis occurred more commonly in the public ICU.

### Mortality and predictors

Overall, 30-day mortality was 10.2%. Crude mortality was higher in the public ICU but did not reach statistical significance. On multivariate logistic regression analysis (Table 2), admission to the public ICU remained independently associated with increased odds of 30-day mortality compared with the private ICU (adjusted OR 4.05, 95% CI 1.179–9.15; *p* = 0.001), after adjustment for age, sex, APACHE II score and inotropic support. Higher APACHE II score (adjusted OR 1.12 per point increase, 95% CI 1.09–1.19; *p* < 0.001) and the requirement for inotropic support (adjusted OR 1.12 per point increase, 95% CI 2.74–20.80; *p* < 0.001) were also independent predictors of mortality.

**TABLE 2:** Multivariable logistic regression identifying independent predictors of mortality.

Variable	Adjusted OR	95% CI	*p*-value
Age (years)	1.01	0.99–1.04	0.308
Male gender	0.36	0.13–0.98	0.045
Public ICU (vs private ICU)	4.05	1.79–9.15	0.001
APACHE II score	1.12	1.06–1.19	< 0.001
Inotropic support	7.55	2.74–20.80	< 0.001

Note: Odds ratios (ORs) are adjusted for all variables included in the multivariate model. Confidence intervals (CI) are reported at the 95% level.

APACHE II, acute, physiological chronic health evaluation II; ICU, intensive care unit.

## Discussion

This study provides a contemporary comparison of trauma ICU outcomes across South Africa’s dual healthcare system and offers several important insights relevant to trauma critical care in LMICs. Both participating institutions are Level I trauma centres, providing comprehensive, consultant-led trauma services with immediate access to specialist surgical care, advanced imaging, operative intervention and critical care facilities. Although they function within different health care sectors, both centres manage high-acuity trauma patients and are staffed by dedicated trauma surgeons who provide both surgical and critical care management. Importantly, neither ICU employs dedicated intensivists, with trauma surgeons fulfilling the role of primary intensivist throughout the patient’s ICU admission. This was an important consideration in the study design, as it minimised the potential confounding effect of differing intensivist involvement and allowed a more direct comparison between the public and private trauma ICU systems.

Despite substantial differences in patient demographics, injury mechanisms and severity profiles, crude 30-day mortality did not differ significantly between the public and private trauma ICUs analysed in this study. However, given the relatively small number of mortality events, the study may have been underpowered to detect modest but clinically meaningful differences between groups. The findings of this study suggest that illness severity and physiological derangement are the principal drivers of short-term mortality following severe trauma, irrespective of healthcare sector.

The predominance of penetrating trauma among younger male patients in the public sector reflects the ongoing burden of interpersonal violence in urban South Africa, whereas the older age and higher prevalence of blunt trauma in the private sector likely reflect differing referral patterns and comorbidity profiles. The higher APACHE II and ISS values observed in the private ICU indicate that this cohort presented with greater overall physiological and anatomical insult, yet without a corresponding increase in mortality.

Differences in ICU admission criteria should also be considered when interpreting these findings. Admission to the public trauma ICU is primarily reserved for patients requiring organ support because of limited critical care capacity, resulting in a highly selected cohort of critically ill patients. The admission criteria for the private ICU differ and may reflect greater bed availability. These differences in case selection may contribute to the variation in patient characteristics observed between sectors and should be considered when comparing outcomes.

Although crude mortality was similar between sectors, admission to the public ICU remained independently associated with mortality on multivariate analysis. This finding may reflect unmeasured system-level factors such as delays in access to definitive care, inter-facility transfer patterns, ICU bed availability or differences in post-operative monitoring and support services that are not fully captured by traditional injury severity scoring systems. Emerging South African evidence suggests that trauma outcomes are influenced not only by injury, but also by health-system factors including delays to definitive care, referral pathways and access to specialist resources, supporting a system-based interpretation of trauma mortality.^12^

While overall mortality was comparable between sectors, the higher incidence of nosocomial sepsis in the public ICU warrants consideration. This finding is likely multifactorial, and it is hypothesised that differences in patient volume, staffing ratios, limited isolation facilities, challenges in timely source control, variability in access to infection prevention resources and infrastructure may play a role.

Importantly, the absence of a marked mortality difference despite disparate resource availability underscores the resilience, adaptability and expertise of multidisciplinary trauma teams within the public sector. At the same time, these findings highlight opportunities for system-level interventions, principally infection prevention, ICU staffing and critical care capacity, that can potentially yield incremental improvements in outcomes.

These findings underscore the complexity of trauma system performance within dual healthcare systems. While the private sector may have greater access to advanced resources, public sector trauma centres frequently manage substantially higher patient volumes and more penetrating injuries, often under significant resource constraints. Understanding how these systems achieve comparable short-term outcomes may offer important lessons for trauma system design in other middle-income countries facing similar disparities in healthcare infrastructure.

From a global surgery perspective, these findings reinforce that validated severity scoring systems such as APACHE II and ISS retain prognostic utility across healthcare sectors in LMIC settings. However, reliance on mortality alone may underestimate sector-specific differences in quality of care. Future studies should incorporate longer-term outcomes, functional recovery and post-ICU morbidity to more fully characterise the impact of healthcare system disparities.

### Limitations

This study has several limitations. Its retrospective design is susceptible to selection bias, information bias and residual confounding. As only two Level I trauma centres within a single metropolitan area were included, these findings should not be considered representative of public and private healthcare sectors throughout South Africa. Differences in institutional practices, referral pathways, staffing models and resource allocation across other centres may limit the generalisability of these results. Furthermore, the relatively small sample size and low mortality rate limited statistical power to detect modest differences between groups. Finally, the chronic health component of the APACHE II score and individual patient comorbidities were not evaluated.

### Future directions

Prospective, multicentre studies incorporating process-of-care metrics and longer-term outcomes are needed to guide trauma ICU system strengthening in LMICs.

## Conclusion

In this dual-sector trauma ICU cohort, marked differences in demographics, injury patterns and severity profiles were observed between public and private healthcare sectors. Despite these differences, short-term mortality did not differ significantly, with illness severity emerging as the dominant determinant of outcome. These findings suggest that high-quality trauma critical care can be delivered across sectors while emphasising the importance of system-level factors that may further enhance patient outcomes.
